# Infectivity and Drug Susceptibility Profiling of Different *Leishmania*-Host Cell Combinations

**DOI:** 10.3390/pathogens9050393

**Published:** 2020-05-20

**Authors:** Kyung-Hwa Baek, Laura Piel, Thibault Rosazza, Eric Prina, Gerald F. Späth, Joo Hwan No

**Affiliations:** 1 Leishmania Research Laboratory, Institut Pasteur Korea, Seongnam-si, Gyeonggi-do 13488, Korea; kyunghwa.baek@ip-korea.org; 2 Institut Pasteur, Unité de Parasitologie Moléculaire et Signalisation, 75015 Paris, France; laura.piel@pasteur.fr (L.P.); thibault.rosazza@gmail.com (T.R.); eric.prina@pasteur.fr (E.P.); gerald.spaeth@pasteur.fr (G.F.S.)

**Keywords:** *Leishmania amazonensis*, *Leishmania donovani*, primary bone marrow-derived macrophage, THP-1, drugs, susceptibility

## Abstract

Protozoan parasites of the genus *Leishmania* are the causative agents of leishmaniasis, a spectrum of a disease that threatens public health worldwide. Although next-generation therapeutics are urgently needed, the early stage of the drug discovery process is hampered by very low hit rates from intracellular *Leishmania* phenotypic high-throughput screenings. Designing and applying a physiologically relevant in vitro assay is therefore in high demand. In this study, we characterized the infectivity, morphology, and drug susceptibility of different *Leishmania* and host cell infection combinations. Primary bone marrow-derived macrophage (BMDM) and differentiated human acute monocytic leukemia (THP-1) cells were infected with amastigote or promastigote forms of *Leishmania amazonensis* and *Leishmania donovani*. Regardless of host cell types, amastigotes were generally well phagocytosed and showed high infectivity, whereas promastigotes, especially those of *L. donovani*, had predominantly remained in the extracellular space. In the drug susceptibility test, miltefosine and sodium stibogluconate (SSG) showed varying ranges of activity with 14 and >10-fold differences in susceptibility, depending on the host-parasite pairs, indicating the importance of assay conditions for evaluating antileishmanial activity. Overall, our results suggest that combinations of *Leishmania* species, infection forms, and host cells must be carefully optimized to evaluate the activity of potential therapeutic compounds against *Leishmania*.

## 1. Introduction

Leishmaniasis is a neglected disease transmitted via phlebotomine sandflies (Diptera: Psychodidae) infected with protozoan parasites from the genus *Leishmania*. Visceral leishmaniasis (VL), also known as kala-azar, is the most fatal form of the disease, characterized by high fever, hepatosplenomegaly, and pancytopenia, whereas the most common form, cutaneous leishmaniasis (CL), causes ulcerative skin lesions [1,2,3]. Collectively, over one billion people are at risk of leishmaniasis, with an incidence of 0.7 to 1 million new cases annually across 100 countries including those in Asia, East Africa, South America, and the Mediterranean region [4]. Climate change, population mobility, and socioeconomic conditions are important factors that affect the global distribution of the diseases [4,5]. With no effective vaccine currently available, chemotherapy is the only mechanism to address leishmaniasis. However, current treatments are limited, consisting of sodium stibogluconate (SSG), amphotericin B (AmpB), paromomycin, and miltefosine [6,7,8,9,10], and these treatments have limitations such as severe side effects [11], high costs, complicated administration routes [12], long treatment courses, and regimental variation in different countries [13].

To deliver drugs that effectively treat *Leishmania* with lower adverse effects and higher accessibility, several organizations, including the Drugs for Neglected Diseases *initiative* (DND*i*), have started fueling the global *Leishmania* drug pipeline with new potential chemotherapeutics. DND*i*-6148 and DND*i*-0690 are in phase I clinical trials, and several others are undergoing preclinical studies [14,15], following development from a phenotypic approach. Furthermore, with the absence of a clinically validated *Leishmania* drug target, a cell-based approach is regarded as an efficient way to fill in the pipeline.

Many different phenotypic assays have been developed and adapted to high-throughput screening (HTS) systems. The simplest assay is the exposure of promastigotes to screening compounds, followed by measuring their viability. This assay of using the insect form of the parasite is easy, fast, and robust, but selected compounds may not be effective on intracellular amastigotes that proliferate in the host macrophage [16]. Alternatively, axenic amastigotes can be used for the viability assay. These axenically cultured amastigotes transformed from promastigotes have amastigote-like morphology but were shown to have different protein expression and drug susceptibility profiles compared to intracellular amastigotes [17,18]. Lastly, bona fide amastigotes developing inside *Leishmania*-infected host cells are widely used for susceptibility assays; favored cell lines for these assays include THP-1, U937, J774.1, and RAW263.7 or primary cells such as bone marrow-derived macrophages (BMDM) and peritoneal macrophages [19,20]. However, drug susceptibility is dependent on host cell type, and, given the slow or fast replication of intracellular *Leishmania* infections depending on their host cells in vitro, these assays may not reflect normal disease progression in an infected human patient. However, with advances in high-content screening, the intracellular model has become a reliable means to search for starting hits or target molecules through high-throughput screening [21,22,23].

In vitro infection and intracellular multiplication of *Leishmania donovani* parasites using hamster peritoneal macrophages was first characterized by Chang et al. in the mid-1970s [24,25]. A few years later, several researchers reported the activity of antileishmanial compounds against intracellular *L. donovani* using a mouse peritoneal macrophage as a host cell [26,27]. Importantly, the difference of antileishmanial drug susceptibility between extracellular and intracellular *Leishmania* has been characterized by the emphasis of the use of the latter form for activity evaluations [28]. With limited supplies of fresh primary macrophages in common laboratory settings, Gebre-Hiwot et al. reported the use of THP-1 cell line as a host for in vitro drug screening of intracellular *Leishmania* [29]. More recently, based on the ability to culture the host cell in a large scale using cell lines, several groups have established intracellular *Leishmania* assays into HTS systems to identify a large number of small molecule and natural product inhibitors for antileishmanial drug discovery [21,22,30,31,32,33,34]. Even though such HTS systems have exponentially increase the number of compounds tested against intracellular *Leishmania*, the differences of compound susceptibility based on the host cell type and the parasite strain (including clinical isolates) was reported, advocating the importance of validating the drug activity in different conditions and harmonizing *Leishmania* drug evaluation assays to standardize the method for monitoring drug susceptibility [35,36,37].

**Figure 1:**
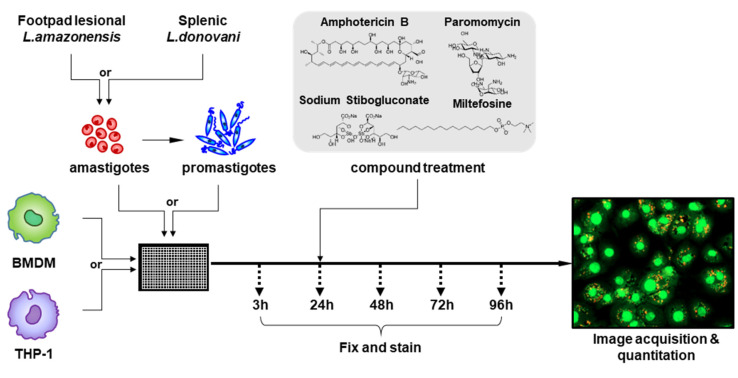
Schematic overview of intracellular *Leishmania* assay. The host cells (BMDM or THP-1) were plated and differentiated, then infected by *Leishmania amazonensis* amastigotes isolated from footpads of BALB/c mice, *Leishmania donovani* amastigotes from hamster spleens, or promastigotes transformed from amastigotes of each species with a multiplicity of infection (MOI) of 1:5, 1:10 or 1:20. Three, 24, 48, 72, and 96 h post-infection (hpi), cells were fixed and stained with Draq-5 fluorescent probe and visually analyzed. For drug testing, reference compounds were added at 24 hpi.

Based on the current needs to standardize intracellular *Leishmania* assays, in this study, we systematically revisited the factors that influence infection and drug susceptibility such as host cell type, parasite species, parasite life stage, and multiplicity of infection (MOI). We utilized an eight-way (2 × 2 × 2) design: BMDM and THP-1 were selected as host cells, and *Leishmania* (*Leishmania*) *amazonensis* (*L. amazonensis*) and *Leishmania* (*Leishmania*) *donovani* (*L. donovani*) were selected as models for CL and VL, respectively, using both amastigote and promastigote forms (Figure 1). At MOIs of 1:5, 1:10, and 1:20, images were acquired between 3 and 96 h post-infection (hpi), followed by quantitative and morphological analysis. Furthermore, susceptibility to commonly used antileishmanial drugs, AmpB, miltefosine, SSG, and paromomycin, was evaluated in all eight combinations of host–parasite pairs to compare their efficacies.

## 2. Results

### 2.1. Infection of BMDM with Leishmania spp.

**Figure 2:**
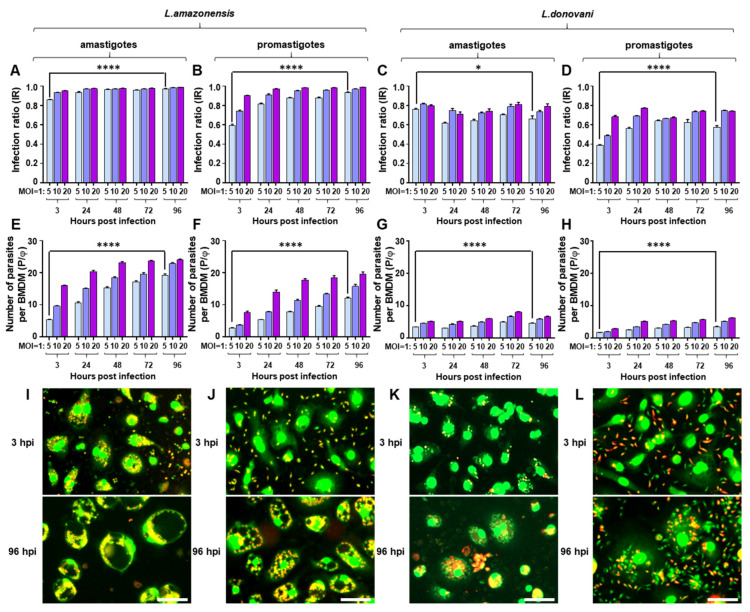
Comparison of infection parameters and morphology of BMDM infected with *Leishmania* spp. (**A**) infection ratio (IR), (**E**) the number of parasites per macrophage (P/φ), and (**I**) phenotypic images of *L. amazonensis* amastigote infection. (**B**) IR, (**F**) P/φ, and (**J**) phenotypic images of *L. amazonensis* promastigote infection. (**C**) IR, (**G**) P/φ, and (**K**) phenotypic images of *L. donovani* amastigote infection. (**D**) IR, (**H**) P/φ, and (**L**) phenotypic images of *L. donovani* promastigote infection. Phenotypic images are from an MOI of 1:10 at 3 and 96 h post-infection (scale bar, 50 µm). Results represent the values with standard deviations from quadruplicate measurements. The significance of differences between groups was calculated by Student’s *t*-test using GraphPad Prism (Version 6.0). * *p* < 0.05, **** *p* < 0.0001.

In mouse BMDM cells infected with *L. amazonensis* amastigotes, infection ratios (IRs) in the early phase of infection (3 hpi) were as high as 0.857, 0.934, and 0.951 with MOIs of 1:5, 1:10, and 1:20, respectively. With minimal increase, all the values reached over 0.97 at 96 hpi (Figure 2A, Supplementary Table S1). However, for the average number of parasites per BMDM (P/φ), a dramatic increase in the values was observed during the measured time points. For an instance, with an MOI of 1:5, P/φ, the value was 5.37 at 3 hpi and gradually increased to reach 19.2 at 96 hpi (Figure 2E). The values also increased at higher MOIs until they reached 20–23 amastigotes per host cell. In phenotypic observation, only a few extracellular parasites were found at 3 and 24 hpi, and small parasitophorous vacuoles (PVs) appeared enlarged afterwards, with the parasites showing the typical location at the inner edge of the PVs (Figure 2I and Supplementary Figure S1). In *L. amazonensis* promastigote infection, IR and P/φ values were lower than the amastigote infection at 3 hpi but gradually increased until 96 hpi (Figure 2B,F). For instance, at an MOI of 1:5, the 3 hpi IR was 0.594 but increased by 57.6% to 0.936 at 96 hpi (Supplementary Table S1). Given that a large number of parasites were observed extracellularly at 3 hpi (Figure 2J), these promastigotes may have had slow infection kinetics. In BMDM infected with splenic amastigotes of *L. donovani*, a high IR (0.7–0.8) was present in all MOIs after only 3 hpi; this IR was maintained until 96 hpi. P/φ was between 3–5 until 48 hpi and peaked at 72 hpi. (Figure 2C,G). As in *L. amazonensis*, most of the amastigotes infected BMDM by 3 hpi (Figure 2K); however, no prominently visible PVs were observed at any time point as previously reported [38]. In promastigote infections, the IR at an MOI of 1:5 was 0.388, half that of amastigote infections, and even lower IRs were observed at higher MOIs (Figure 2D). IR increased until 24 hpi and was maintained thereafter. No significant differences were observed between promastigote and amastigote infection P/φ values in which the values were lower with no increments (Figure 2G,H). Extracellular promastigotes were observed even after 96 hpi, but these parasites presumably increased IR until only 24 or 48 hpi, depending on the MOI. In the images, no distinctively visible PVs were observed throughout the time points as in the case of *L. amazonensis* (Figure 2L).

### 2.2. Infection of Differentiated THP-1 with Leishmania spp. and Comparison to BMDM Infection

**Figure 3:**
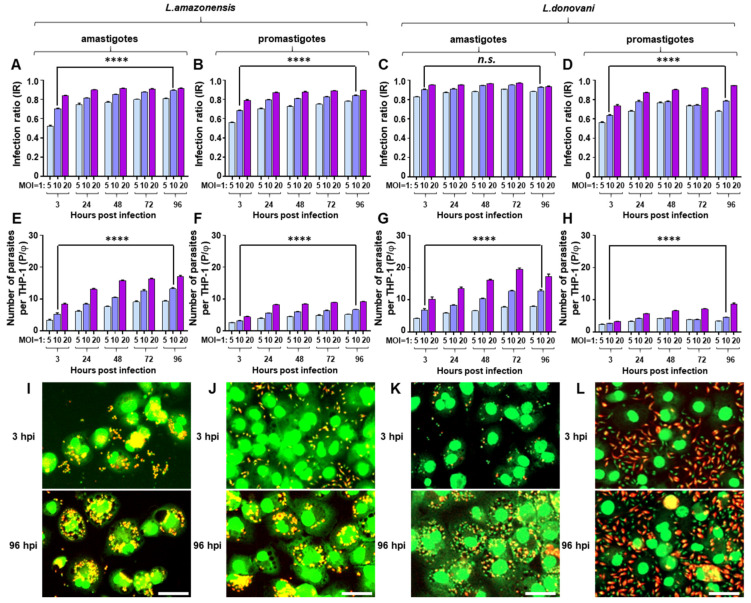
Comparison of infection parameters and morphology of differentiated THP-1 infected with *Leishmania* spp. (**A**) infection ratio (IR), (**E**) the number of parasites per macrophage (P/φ), and (**I**) phenotypic images of *L. amazonensis* amastigote infection. (**B**) IR, (**F**) P/φ, and (**J**) phenotypic images of *L. amazonensis* promastigote infection. (**C**) IR, (**G**) P/φ, and (**K**) phenotypic images of *L. donovani* amastigote infection. (**D**) IR, (**H**) P/φ, and (**L**) phenotypic images of *L. donovani* promastigote infection. Phenotypic images are from an MOI of 1:10 at 3 and 96 h post-infection (scale bar, 50 µm). Results represent the values with standard deviations from quadruplicate measurements. The significance of differences between groups was calculated by Student’s *t*-test using GraphPad Prism (Version 6.0). **** *p* < 0.0001, *n.s*; not significance of difference.

In the PMA-differentiated THP-1 macrophage model, IRs were lower than BMDM infected with *L. amazonenesis* but slightly higher with *L. donovani* infection (Figure 3). For example, *L. amazonensis* amastigote infection of THP-1 at 3 hpi with a 1:10 MOI had 0.522 of IR which is 39.1% lower than that of BMDM (IR = 0.857), but with *L. donovani*, IR increased by 8.12% in the same infection condition. After 93 h (at 96 hpi), all the infections at an MOI of 1:10 reached around or over 0.8 showing generally high infection capacity regardless of parasite species. In terms of P/φ, a similar magnitude of increase from 3 hpi to 96 hpi was observed for both *L. amazonensis* and *L. donovani* amastigotes infections. For instance, P/φ increased from 5.25 to 13.2 for *L. amazonensis*, and from 6.87 to 12.6 for *L. donovani* amastigote infections at an MOI of 1:10. These P/φ values coincide with previous reports that intracellular parasites increase in *L. donovani* axenic amastigote infection [39]. For the promastigote infection, both species showed extracellular parasites when exposed to THP-1 at 3 hpi, but similar to BMDM, *L. amazonensis* quickly and completely infect the host whereas, with *L. donovani*, the parasites infected the host cell to some extent as shown in Figure 3D,H, but still a large number of extracellular promastigotes were observed at 96 hpi (Figure 3J,L). Even with existence of these extracellular parasites, IR nor P/φ did not increase dramatically, meaning only a minimal continuous infection occurred. In morphological observations, relatively smaller sized PVs were observed with *L. amazonensis* infections compare to that of BMDM infections (Figure 3I,J). For *L. donovani*, PVs were only seen with amastigotes infected THP-1 which was not even observed in BMDM (Figure 3K,L).

### 2.3. Comparison of IR and P/φ between the Assays

**Figure 4:**
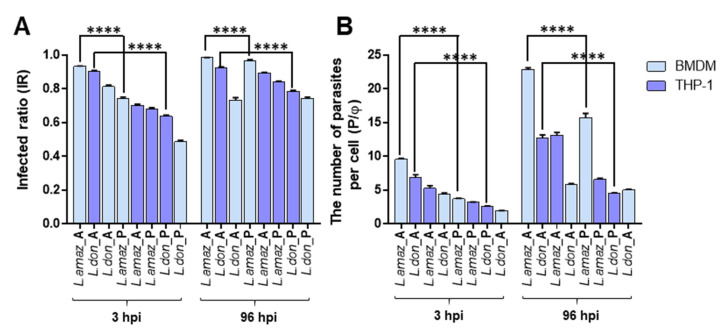
Comparison of infection parameters of bone marrow-derived macrophages (BMDM) or THP-1 infected with *Leishmania* amastigotes or promastigotes (MOI of 1:10) at 3 and 96 h post-infection (hpi). (**A**) Infection ratio (IR) and (**B**) the number of parasites per macrophage (P/φ). Results represent the values with standard deviations from quadruplicate measurements. The significance of differences between groups was calculated by Student’s *t*-test using GraphPad Prism (Version 6.0). **** *p* < 0.0001 (*L. amaz* = *L. amazonensis*, *L. don* = *L. donovani*, A = amastigote, and P = promastigote).

To compare the investigated infection systems side-by-side, IR and P/φ values at an MOI of 1:10 were plotted in the same axis. At 3 hpi, infections with amastigotes showed relatively high IR values relative to promastigote infections (Figure 4A, Supplementary Table S1). After 96 hpi, all systems exceeded an IR of 0.73, and especially for promastigote infections prominent increases were seen, possibly due to its slow infection kinetics. In terms of P/φ, there were several infection conditions with notable increases. *L. amazonensis* amastigote infections to BMDM showed significant increase in P/φ value from 9.56 to 22.9 (2.40 fold) with an MOI of 1:10 which was even higher (3.58-fold) at a lower MOI of 1:5. Furthermore, the infection with promastigote displayed a 4.22-fold increase (from 3.72 to 15.7), but this is likely to be a combination of slow infecting extracellular parasites and replication of internalized amastigotes (Figure 4B, Supplementary Table S2). For *L. donovani*, infection of THP-1 with amastigote form showed 1.83-fold increase in the value of P/φ (from 8.87 to 12.6), which was not the case in BMDM infection amastigotes (Figure 4B, Supplementary Table S2).

### 2.4. Drug Susceptibility

**Figure 5:**
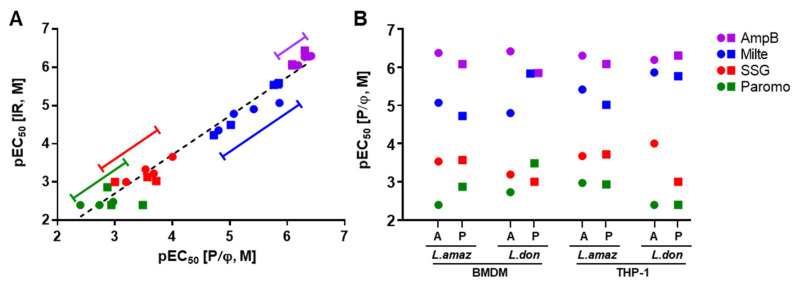
Drug potencies (pEC_50_) derived from eight different host cell–*Leishmania* infection combinations. (**A**) Infection ratio (IR) versus the number of parasites per macrophage (P/φ)-based analysis used for pEC_50_ calculation. (**B**) Comparative plotting of pEC_50_ values with statistical averages (circle = amastigote, square = promastigote). pEC_50_ equal to −logEC_50_ and [M] indicates the unit is in molar concentration of mole/liter. (*L. amaz* = *L. amazonensis*, *L. don* = *L. donovani*, A = amastigote, and P = promastigote).

In order to evaluate susceptibility to compounds in the infection systems, we selected drugs used in clinics (AmpB, miltefosine, SSG, and paromomycin) and tested them in all eight host–parasite infection pairs. For all the assays, an MOI of 1:10 was used since sufficient infection windows (IR < 0.7 and P/φ < 5) were observed in most of infection conditions. EC_50_ values were calculated using IR or parasite numbers to check for differences due to the extracted parameters from the images (Supplementary Figures S2–S5). The activity measure between IR and P/φ did not differ significantly among drugs tested in different systems (R^2^ = 0.96 and slope = 1.01, Figure 5A). In terms of general activity of compounds based on the average EC_50_ values of all the tested systems, the drugs showed the following sequence of potency based on IR and P/φ respectively: AmpB > miltefosine > SSG > paromomycin (Table 1, Supplementary Table S3). Overall, *L. amazonensis* are more sensitive than *L. donovani* and parasites from the same species are more susceptible when they are hosted in THP-1 compared to BMDM. Among the drugs, a large range of variation was seen in miltefosine and SSG (Figure 5A). 

Sensitivity to tested drugs among infection pairs was generally divided into two groups, from more to less sensitive with some variation; THP-1 (*L. amazonensis* amastigote) ≈ THP-1 (*L. donovani amastigote*) ≈ BMDM (*L. amazonensis* amastigote) > THP-1 (*L. amazonensis* promastigote) ≈ BMDM (*L. amazonensis* promastigote) ≈ BMDM (*L. donovani* amastigote). Drug sensitivity was generally higher in *L. amazonensis* amastigote infections of THP-1 host cells (Figure 5B). Among the tested drugs, SSG, which is known to involve host cellular mechanism for its activity, showed low potency in the systems with *L. donovani* promastigote infection. Since a large number of extracellular *L. donovani* promastigotes unaffected by SSG was present during the incubation period, a continuous infection by those parasites may have led to high IR and P/φ causing low susceptibility to SSG.

#### 2.4.1. AmpB Susceptibility

**Figure 6:**
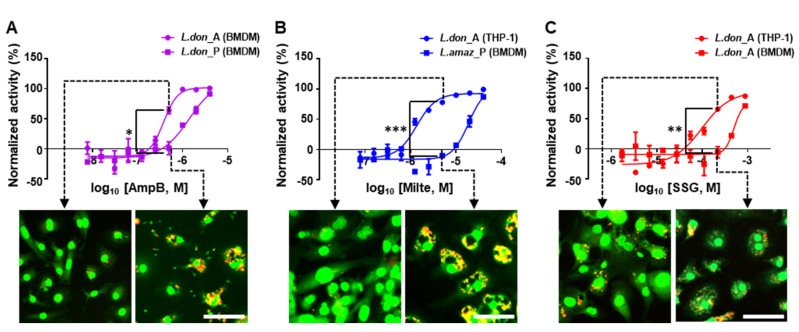
Dose-response curves and corresponding images of *Leishmania*-infected host cells treated with reference drugs demonstrating differences in susceptibility. (**A**) *L. donovani* amastigote-infected and promastigote-infected BMDM exposed to AmpB, (**B**) *L. donovani* amastigote-infected THP-1 and *L. amazonensis* promastigote-infected BMDM exposed to miltefosine, or (**C**) *L. donovani* amastigote-infected THP-1 and BMDM exposed to SSG (scale bar, 50 µm). (*L. amaz* = *L. amazonensis*, *L. don* = *L. donovani*, A = amastigote, and P = promastigote).

The average EC_50_ value across all tested conditions was 0.68 µM and the least magnitude of variation was observed (Table 1, Figure 5A,B). BMDM infection by *L. donovani* amastigotes showed the highest susceptibility to AmpB; we observed a 3.7-fold difference relative to the least susceptible BMDM infection by the same parasites but in promastigote form (EC_50_ = 0.38 µM and 1.4 µM, respectively; Figure 6A). Except for the least susceptible combination, EC_50_ values of each pair exhibited activities within two-fold differences of one another (EC_50_ = 0.42 to 0.82 µM), showing consistent activity regardless of infection conditions.

#### 2.4.2. Miltefosine Susceptibility

**Figure 7:**
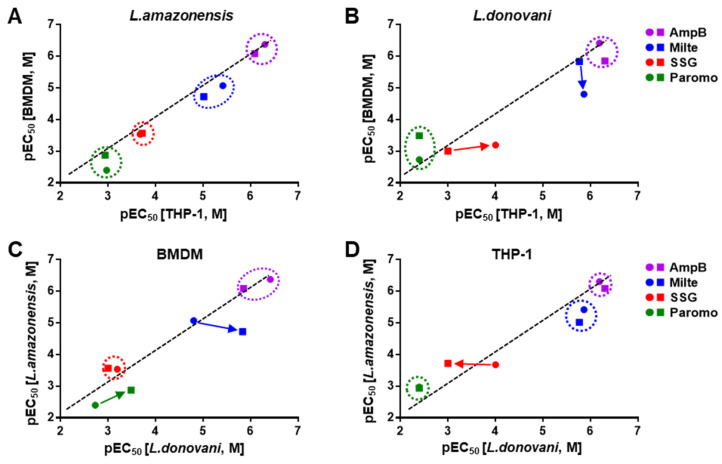
Comparison of (**A**) pEC_50_ of *L. amazonensis* infection of bone marrow-derived macrophages (BMDM) versus THP-1, (**B**) pEC_50_ of *L. donovani* infection of BMDM versus THP-1, (**C**) pEC_50_ of *L. amazonensis* versus *L. donovani*-infected BMDM, (**D**) pEC_50_ of *L. amazonensis* versus *L. donovani*-infected THP-1 with treatment of AmpB, miltefosine, SSG, and paromomycin. Results represent the EC_50_ values with standard deviations from duplicate measurements (circle = amastigote, square = promastigote). pEC_50_ equal to -logEC_50_ and [M] indicates the unit is in molar concentration of mole/liter.

Miltefosine showed an average EC_50_ of 7.63 µM and the variation of EC_50_ values was highest among the tested drugs (Table 1, Figure 5A,B). The pair most susceptible to miltefosine was THP-1 infected by *L. donovani* amastigotes (EC_50_ = 1.37 µM) and the least susceptible was BMDM infected by *L. amazonensis* promastigotes (EC_50_ = 18.9 µM), which showed a 14-fold difference (Figure 6B). Within the infections of BMDM by *L. donovani*, simply changing the infection form of parasite from promastigote to amastigote shifted the EC_50_ from 1.47 to 15.7 µM, an 11-fold difference (Figure 7B,C). Activity was generally more potent against the parasites infecting THP-1 than BMDM, and in terms of species, *L. donovani* was relatively more susceptible to miltefosine compared to *L. amazonenesis*.

#### 2.4.3. SSG Susceptibility

In general, SSG susceptibility was poor, with EC_50_ values averaging above 463 µM (Table 1). Infections with *L. amazonenesis* showed relatively less variation of EC_50_ values (from 191 to 292 µM) regardless of the host cell type and the form of parasite infections. On the other hand, infections with *L. donovani* showed varying SSG activity depending on the condition (Figure 7B,D). For instance, SSG was inactive in the infections of BMDM and THP-1 using promastigote form (>1000 µM), but became active in amastigotes infections with EC_50_ = 98.8 µM for THP-1 and 638 µM for BMDM (Figure 6C). Interestingly, SSG susceptibility was mostly active in systems in which PVs were observed. For example, in *L. donovani*, where SSG had poor efficacy in combinations with promastigote infection which showed no PVs formation, an EC_50_ of 98.9 µM was observed in THP-1 cells infected by amastigotes, a combination that exhibited an unusually large number of small PVs.

## 3. Discussion

In this study, we presented results of in vitro infectivity and drug susceptibility profiles combining different *Leishmania* species, life stages, and host cell types. Amastigotes or promastigotes of cutaneotropic *L. amazonensis* or viscerotropic *L. donovani* were used to infect murine BMDM or differentiated THP-1 cells. Given the complexity of parasite quantification, drug susceptibility assessment, and the experimental model system used, we developed an eight-way comparison of host cells, parasite species, and parasite life stages to compare the infectivity and drug susceptibility of *Leishmania* parasites, which led to several key points. First, infections with amastigotes had higher infectivity and more physiologically relevant phenotypes of parasite replication. In this in vitro study, THP-1 and BMDM infection by *L. amazonensis* amastigote showed not only high IR but also a significant increase in intracellular parasite number. A significantly dramatic increase is seen when BMDM is infected at a low MOI of 1:5. It is important to note that the incubation temperature at 34 °C, reflecting a cutaneous environment, is crucial for the amastigote replication [40]. THP-1 infection by *L. donovani* amastigote also showed high IR and an increase in parasite number in kinetic experiments. However, in promastigote infections, except for the *L. amazonensis*–BMDM combination, extracellularly replicating promastigotes did not lead to high IR or an increase in intracellular parasite number. A likely explanation is that there are less portion of infectious metacyclic promastigotes (a larger portion of replicating procyclics) and some of promastigotes are ingested and degraded at the same time. Second, infections with *L. amazonensis* generally showed formations and enlargements of PVs, the hallmark of *Leishmania* infection [41]. In *L. donovani*, only small and tight PVs were observed in THP-1 infected with amastigote form. These smaller vacuoles were previously reported where a single amastigotes surrounded by a tightly fitted membrane without vacuolar space was mostly observed, with some other harboring two or three amastigotes in relatively larger vacuoles [38]. PVs containing *L. donovani* are suggested as a specialized early endocytic compartments in which the parasite modulates endo-lysosomal pathways by preventing lysosomal transport in macrophages [42]. Taken together with the first point, *L. amazonensis* infections to BMDM and *L. donovani* infections to THP-1 using amastigote form are suggested for general infection related experiments to possibly reflect physiologically relevant condition in vitro. Third, drug susceptibility depends on the host cell–parasite combination. We observed wide variation in drug activity for miltefosine and SSG; lower EC_50_ values do not necessarily mean the assay is properly designed, but when a drug used clinically is not active in vitro, the suitability of the tested system should be questioned. For instance, here, we demonstrated that the miltefosine activity dramatically changed depending on host cell type in which the drug was more active against the parasites in THP-1 than in BMDM. Regardless of *Leishmania* species or host cell types, the general order of drug susceptibility was, from high to low susceptibility, AmpB, miltefosine, SSG, and paromomycin, consistent with susceptibility data previously reported [35,43]. Though it is difficult to draw a direct correlation between the sequences of in vitro potency to the clinical efficacy of each drug due to their pharmacokinetics property and tissue distribution in humans, it is at least worth mentioning that the least potent drug in vitro, paromomycin, showed varying ranges of efficacy in clinical studies [44]. When we consider these three points, we suggest using THP-1 for *L. donovani* infections, and BMDM (or alternatively THP-1) for *L. amazonensis* with amastigotes as the form of parasite.

Antileishmanial assessments of compounds in vitro are performed largely in these different formats: axenic promastigotes, axenic amastigotes and intracellular amastigotes [28,45]. Promastigote is the form surviving inside a sand-fly gut which can be easily cultured in vitro. The viability of promastigote is easily quantified by a colorimetric method such as Alamar Blue or by fluorescence signal of the engineered parasite [46]. Axenic amastigotes result from the transformation of promastigotes in response to an increase in temperature and an acidic pH, and their viability is quantified similarly to the method of promastigote assay [28,47,48]. Axenic amastigote assay can be recommended for the evaluation of compounds which does not involve cellular mechanism, such as amphotericin B and miltefosine, since the parasite exists as an extracellular form. The intracellular amastigote model is considered as “the golden standard” for in vitro drug discovery research including the evaluation of drug resistance of field strains. In this model, host cell-mediated effects are taken into consideration unlike the cases of promastigote or axenic amastigote assays, and the reports of difference of drug susceptibility such as SSG activity compare to extracellular *Leishmania* assays [28]. The method for intracellular *Leishmania* assay varies depending on the type of host cells and the form of the parasites used for infection. In most of methods, the quantification is done by counting the stained or fluorescently engineered parasites, or by reading bioluminescent signal of *Leishmania* expressing luciferase [19,27,29,49,50,51,52]. Many groups have reported differences of compound susceptibilities depending on the method mentioned, and a large portion of inhibitors found active in promastigotes or axenic amastigotes were found not active in intracellular amastigote assay [20,21,22,28,30]. This shows that evaluation of compound activity in physiologically relevant in the in vitro model—i.e., intracellular amastigote assay—represents a key step to successful drug development.

*Leishmania* parasites are known to infect several cells of the immune system, including primary macrophages, monocytes, dendritic cells, neutrophils, and immortalized immune cells [53,54]. A select group of macrophages are widely used for intracellular *Leishmania* assays. The use of primary cells, such as mouse BMDM or peritoneal macrophages, is thought to better mimic in vivo infections, but these cells require time-consuming, complicated protocols for isolation and differentiation [55]. Conversely, cell lines, including J774.1, U937, THP-1, and RAW264.7, can be cultured easily for the infections. For the first three monocytes, cells are differentiated using phorbol-12-myristate-13-acetate (PMA), retinoic acid, macrophage colony-stimulating factor (M-CSF), or 1α-25-dihydroxyvitamine D3 (vD3) [56]. The choice of the host cells for parasite infection and drug screening is not trivial as they exhibit varying range of susceptibility to infection [35]. Indeed, we used BMDM and THP-1 as representatives of primary cells and cell lines to evaluate parasite infectivity and drug susceptibility. Among the choices for cell lines, THP-1 was selected in this study since it is one of widely used host cell for intracellular *Leishmania* assays from small-scale testing to large HTS campaigns, and plus, this cell line is regarded as a multifaceted model for the study of monocyte–macrophage differentiation and immune-modulating effects at various conditions [57]. In terms of parasite species, *L. donovani* MHOM/SD/62/1S-CL2D was used based on its extensive use for VL drug discovery at both in vitro and in vivo levels, and for CL, *L. amazonensis* was selected with a similar reason [19,20,30,52]. In addition, *L. amazonensis* infection in mice presents some unique features and immunoregulatory mechanisms, making it an interesting model for obtaining further knowledge of potential drug targets in this parasite infection [58]. Based the results from the combinations of infection tests and drug susceptibility evaluation using the selected parasites and host cells, the suitable condition for VL in vitro drug testing is infection of THP-1 by ex vivo amastigote at an MOI of 1:10 with 72 h of compound exposure, and for CL, the condition remains the same, except BMDM is preferred as the host cell for infections. Upon a confirmation of compound activity, a further “time-to-kill” assay may provide useful information regarding the kinetics of compound action [59].

In conclusion, the choice of *Leishmania* species and form, and host cell are critical factors that influence the activity of tested compounds. Hampered by low hit rates with high-throughput phenotypic screening, developing an in vitro assay that reflects human infections is a crucial first step for the success of the drug discovery process. Based on our study, we propose in vitro conditions for testing inhibitors against *Leishmania* based on the intracellular parasite growth, host morphology and drug sensitivity. These findings should be of general interest to the antileishmanial development community because the basic factors influencing drug activity provide insights into the establishment of improved intracellular *Leishmania* assays, which will, in turn, lead to identification of potential cures for *Leishmania* infections.

## 4. Materials and Methods

### 4.1. Animals

Female BALB/c mice (5–6 weeks old; body weight 15–20 g) and male Golden Syrian hamsters (5–6 weeks old; body weight 60–80 g) were purchased from Orientbio Inc. (Seongnam-si, Rep. of Korea) and Central Laboratory Animal Inc. (Seoul, Rep. of Korea), respectively. All animal handling and experiments were performed in compliance with the guidelines and principles established by the Korean Animal Protection Law (http://animalrightskorea.org). All protocols for animal experiments were reviewed and approved by the Institutional Animal Care and Use Committee (IACUC, protocol # IPK-16003-3) of the Institut Pasteur Korea.

### 4.2. Parasite Culture in Vitro

*L. amazonensis* strain LV79 (MPRO/BR/1972/M1841) and *L. donovani* strain Ld1S (MHOM/SD/62/1S-CL2D) expressing mCherry were maintained in BALB/c mice and in hamsters, respectively. Promastigotes were maintained at 28 °C in modified M199 culture medium with 20 mM HEPES (Gibco, Waltham, MA, USA), 0.1 mM adenine (Sigma, St. Louis, MO, USA), 0.0005% hemin (Sigma), 0.0001% biotin (Sigma), 0.0002% biopterin (Santa Cruz, Dallas, TX, USA), and 4.62 mM NaHCO_3_ (Sigma), supplemented with 10% heat-inactivated fetal bovine serum (FBS, Gibco), and 1% streptomycin/penicillin (Gibco). The cultures were diluted every 3 (*L. amazonensis*) or 7 (*L. donovani*) days and underwent no more than five passages to avoid generation of genetic variability [60].

### 4.3. Infection of Mice and Isolation of Parasites

Anesthetized, 6-week-old BALB/c mice were infected with 10^7^ promastigotes of *L. amazonensis* injected subcutaneously in the footpad. When injected footpads became swollen 6–8 weeks after infection, the mice were euthanized using CO_2_. Footpads were collected [46] and homogenized in cold phosphate-buffered saline (PBS) after the removal of the skin and necrotic tissues; tissue debris were then removed by centrifugation at 30× *g* for 10 min at 4 °C. The parasites were harvested by centrifugation of the supernatant at 1500× *g* for 10 min at 4 °C, and red blood cells were removed with ammonium-chloride-potassium (ACK) lysing buffer (Gibco). After 3 washes with PBS, the purified amastigotes were collected and used to infect cells or resuspended in M199 culture medium for differentiation into promastigotes.

### 4.4. Infection of Hamsters and Isolation of Parasites

Anesthetized, 5-week-old hamsters were inoculated with 10^8^ metacyclic promastigotes of *L. donovani* by intracardiac injection. The infected hamsters were euthanized using CO_2_ when their weight decreased by 15% to 20%. Their spleens were collected and homogenized in cold PBS, and tissue debris were removed by centrifugation at 130× *g* for 5 min at 4 °C. The parasites were then harvested by centrifugation of the supernatant at 2000× *g* for 10 min at 4 °C. After 3 washes with PBS, the parasites were further isolated by Percoll gradient [17]. After centrifugation at 3500× *g* for 45 min at 15 °C, parasites were collected from the interface of the gradient, then washed three times with PBS. These purified amastigotes were used to infect macrophages or differentiated into promastigotes.

### 4.5. Host Cell Cultures

#### 4.5.1. Human Monocytic Cell Line

THP-1 human monocyte cell line was grown in RPMI-1640 medium (Gibco) supplemented with 10% heat-inactivated fetal bovine serum (Gibco) at 37 °C with 5% CO_2_ in air. For differentiation, THP-1 cells were treated for 3 days with 50 ng/mL of phorbol 12-myristate 13-acetate (PMA).

#### 4.5.2. Mouse Primary Macrophages

BMDMs were obtained from 8–12 week old BALB/c mice as previously described [61]. The euthanized mice were disinfected with 70% ethanol, and femurs and tibia were dissected from the body. Bone marrow cells were collected and resuspended in RPMI-1640 with 75 ng/mL of recombinant mouse CSF-1 (Invitrogen, Waltham, MA, USA), 10% FBS, and 1% streptomycin/penicillin for differentiation to macrophages. Cells were incubated for 6 days in hydrophobic Petri dishes, to which CSF-1 was added at day 3.

### 4.6. Experimental Infections and Drug Susceptibility Assays

A schematic overview of the assay is illustrated in Figure 1. CSF-1-differentiated BMDM or PMA-differentiated THP-1 were plated in 384-well plates. For the promastigote infections, stationary phase promastigotes were prepared by culturing *L. donovani* for 7 days and *L. amazonensis* for 5 days. Host cells were infected with amastigotes or stationary phase promastigotes at MOIs of 1:5, 1:10, or 1:20 for infectivity characterization at 34 °C for *L. amazonensis* or at 37 °C for *L. donovani* with 5% CO_2_ [40]; a 1:10 MOI was used for drug testing. Cells were then fixed with 4% paraformaldehyde (PFA) and stained with a fluorescent probe (Draq-5, Thermo Fisher, Waltham, MA, USA) at 3, 24, 48, 72, and 96 hpi. For the drug susceptibility assays, infected cells were treated with AmpB (Sigma), miltefosine (MCE, Monmouth Junction, NJ, USA), sodium stibogluconate (Calbiochem, St. Louis, MO, USA), or paromomycin (Sigma) at 24 hpi without wash. After 3 days of drug treatment, cells were fixed with 4% PFA, washed with 1× PBS and stained with Draq-5.

### 4.7. Image Acquisition and Analysis

Cell images were acquired by an automated image analyzer (Operetta, Perkin Elmer Technology, Waltham, MA, USA); analysis included at least 1000 cells in five fields per well. For the imaging of infected cells and parasites, Draq-5 and mCherry signal were observed under a 20× air objective. An image analysis algorithm (Columbus, Perkin Elmer Technology, Waltham, MA, USA) was used to detect Draq-5 signal in the nuclei of cells and parasites; mCherry was not included in this analysis because the parasite fluorescence intensity was not uniform, especially at the early stage of infections (3 hpi), whereas Draq-5 intensity was higher and more distinguishable between parasites (less overlaps with sharp signals) as shown in Supplementary Figure S1. In brief, a large-sized nucleus of host cells was first detected using Draq-5 signal and the host cell boundary masking was performed using the low-intensity signals from cytosols (additional feature of Draq-5). Then, the small-sized nucleus signal by Draq-5 was used to identify parasites within the area of the masked host cell (Supplementary Figure S6A–F). Infection ratio (IR) was determined with the value of the number of infected cells divided by the total number of cells, and the average number of parasites per macrophage (P/φ) was defined by the value of the number of parasites divided by the number of infected cells in the acquired image. The values produced by the algorithm were further analyzed using GraphPad Prism 6 (GraphPad Software, San Diego, CA, USA) for graphical representations and half-maximal effective concentration (EC_50_) value determinations.

## Figures and Tables

**Table 1:** Drug susceptibility (EC_50_) against antileishmanial reference drugs in each host cell–parasite pairs; EC_50_ values calculated based on P/φ. Results represent the values with standard deviations from duplicate measurements (ama = amastigote, pro = promastigote, milite = miltefosine, SSG = sodium stibogluconate, paromo = paromomycin).

Host Cell	*Leishmania* for Infection		EC50 Value of Drugs in µM (95% Confidence Intervals)			
	Species	Infection Form	Amp B	Milte	SSG	Paromo
BMDM	*L. amazonensis*	ama	0.420 (0.389–0.453)	8.49 (6.94–10.4)	292 (242–353)	>4000
		pro	0.818 (0.746–0.898)	18.9 (16.1–22.3)	270 (240–305)	1346 (954–1899)
	*L. donovani*	ama	0.381 (0.341–0.425)	15.7 (10.3–24.0)	638 (525–776)	1854 (1385–2481)
		pro	1.40 (0.873–2.25)	1.47 (1.34–1.60)	>1000	325 (244–434)
THP-1	*L. amazonensis*	ama	0.495 (0.450–0.545)	3.82 (3.23–4.51)	211 (176–253)	1069 (931–1228)
		pro	0.815 (0.703–0.944)	9.57 (4.20–21.8)	191 (157–231)	1156 (924–1446)
	*L. donovani*	ama	0.640 (0.547–0.748)	1.37 (1.22–1.53)	98.9 (78.6–124)	>4000
		pro	0.493 (0.325–0.747)	1.71 (1.33–2.21)	>1000	>4000

## Supplementary Materials

The following are available online at https://www.mdpi.com/2076-0817/9/5/393/s1, Figure S1: Enlarged phenotypic images of BMDM infected with *L. amazonensis* amastigote with an MOI of 1:10 at 96 hpi, Figure S2: Dose-response curves (DRCs) of reference drugs from *L. amazonensis*-infected BMDM, Figure S3: DRCs of reference drugs from *L. donovani*-infected BMDM, Figure S4: DRCs of reference drugs from *L. amazonensis*-infected THP-1, Figure S5: DRCs of reference drugs from *L. donovani*-infected THP-1, Figure S6: Image analysis of intracellular *Leishmania* by Columbus software, Table S1: Infection ratio (IR) of host cells infected with *L. amazonensis* or *L. donovani* by MOI, Table S2: The number of parasites per cell (P/φ) of host cells infected with *L. amazonensis* or *L. donovani* by MOI, Table S3: Drug susceptibility (EC_50_) against antileishmanial reference drugs in each host cell–parasite pair; EC_50_ values calculated based on IR.
