# Supplementary material for: Infectivity and Drug Susceptibility Profiling of Different Leishmania-Host Cell Combinations
Source: Pathogens. 2020 May 20;9(5):393. doi: 10.3390/pathogens9050393 (PMC7281264; doi:10.3390/pathogens9050393)
Supplement: Supplementary file 1 [file pathogens-09-00393-s001.zip › LeishHostDrug_Sup Figure_Revision.PPTX]

## Slide 1
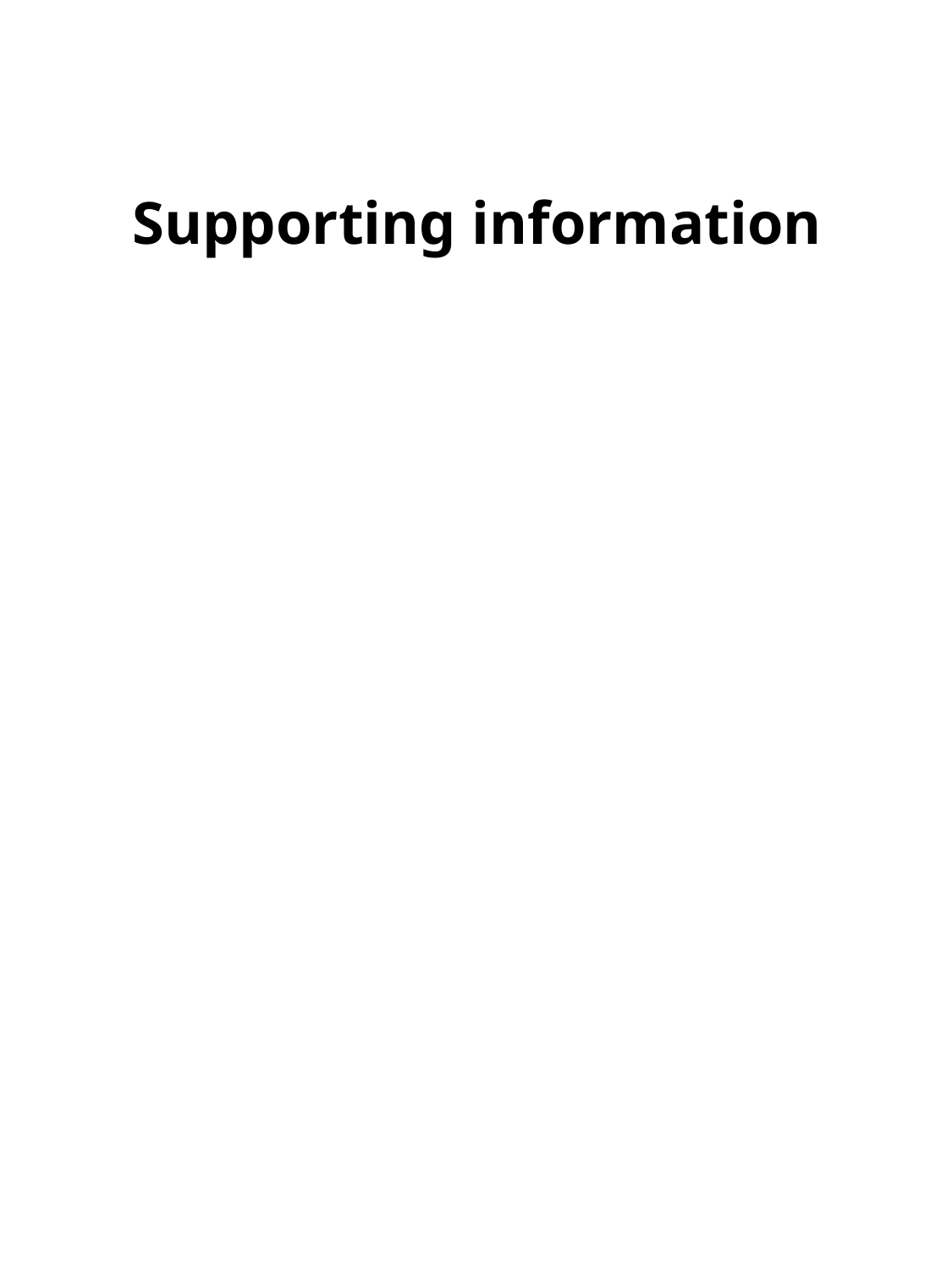

# Supporting information

## Slide 2
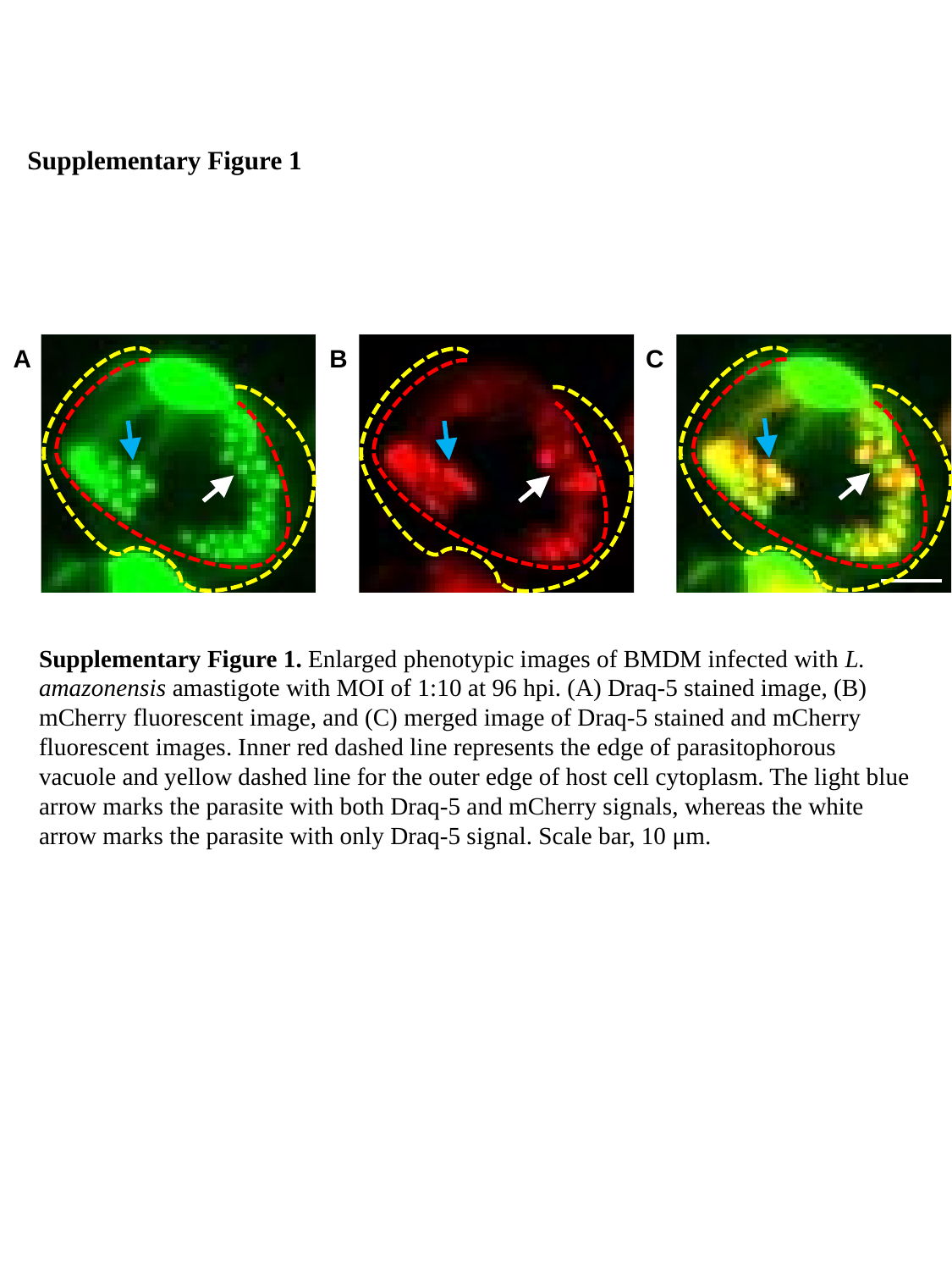

Supplementary Figure 1
A
B
C
Supplementary Figure 1. Enlarged phenotypic images of BMDM infected with L. amazonensis amastigote with MOI of 1:10 at 96 hpi. (A) Draq-5 stained image, (B) mCherry fluorescent image, and (C) merged image of Draq-5 stained and mCherry fluorescent images. Inner red dashed line represents the edge of parasitophorous vacuole and yellow dashed line for the outer edge of host cell cytoplasm. The light blue arrow marks the parasite with both Draq-5 and mCherry signals, whereas the white arrow marks the parasite with only Draq-5 signal. Scale bar, 10 μm.

## Slide 3
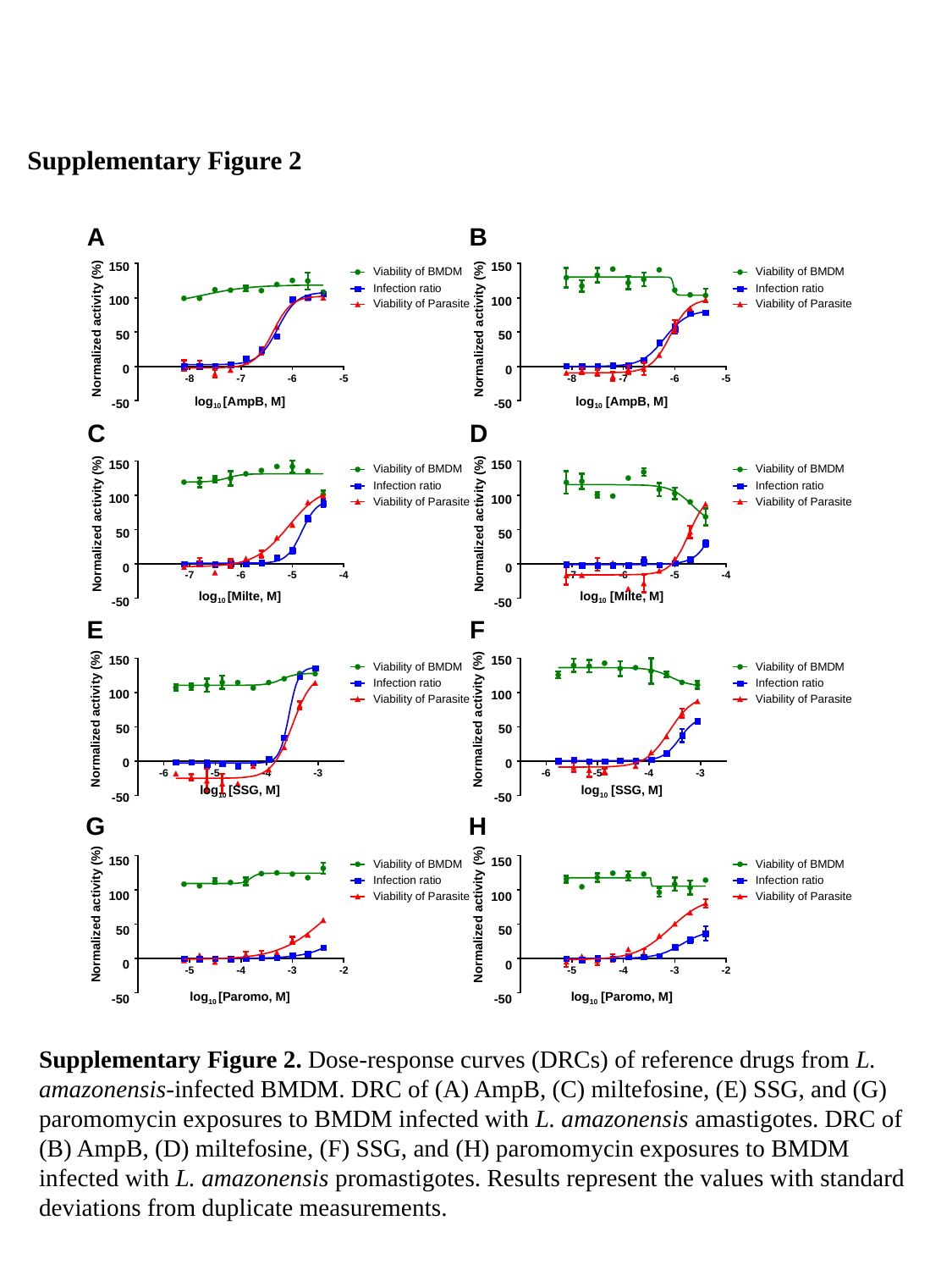

Supplementary Figure 2
A
B
150
100
50
0
-50
150
100
50
0
-50
Normalized activity (%)
Normalized activity (%)
log10 [AmpB, M]
log10 [AmpB, M]
C
D
150
100
50
0
-50
150
100
50
0
-50
Normalized activity (%)
Normalized activity (%)
log10 [Milte, M]
log10 [Milte, M]
E
F
150
100
50
0
-50
150
100
50
0
-50
Normalized activity (%)
Normalized activity (%)
log10 [SSG, M]
log10 [SSG, M]
G
H
150
100
50
0
-50
150
100
50
0
-50
Normalized activity (%)
Normalized activity (%)
log10 [Paromo, M]
log10 [Paromo, M]
Supplementary Figure 2. Dose-response curves (DRCs) of reference drugs from L. amazonensis-infected BMDM. DRC of (A) AmpB, (C) miltefosine, (E) SSG, and (G) paromomycin exposures to BMDM infected with L. amazonensis amastigotes. DRC of (B) AmpB, (D) miltefosine, (F) SSG, and (H) paromomycin exposures to BMDM infected with L. amazonensis promastigotes. Results represent the values with standard deviations from duplicate measurements.

## Slide 4
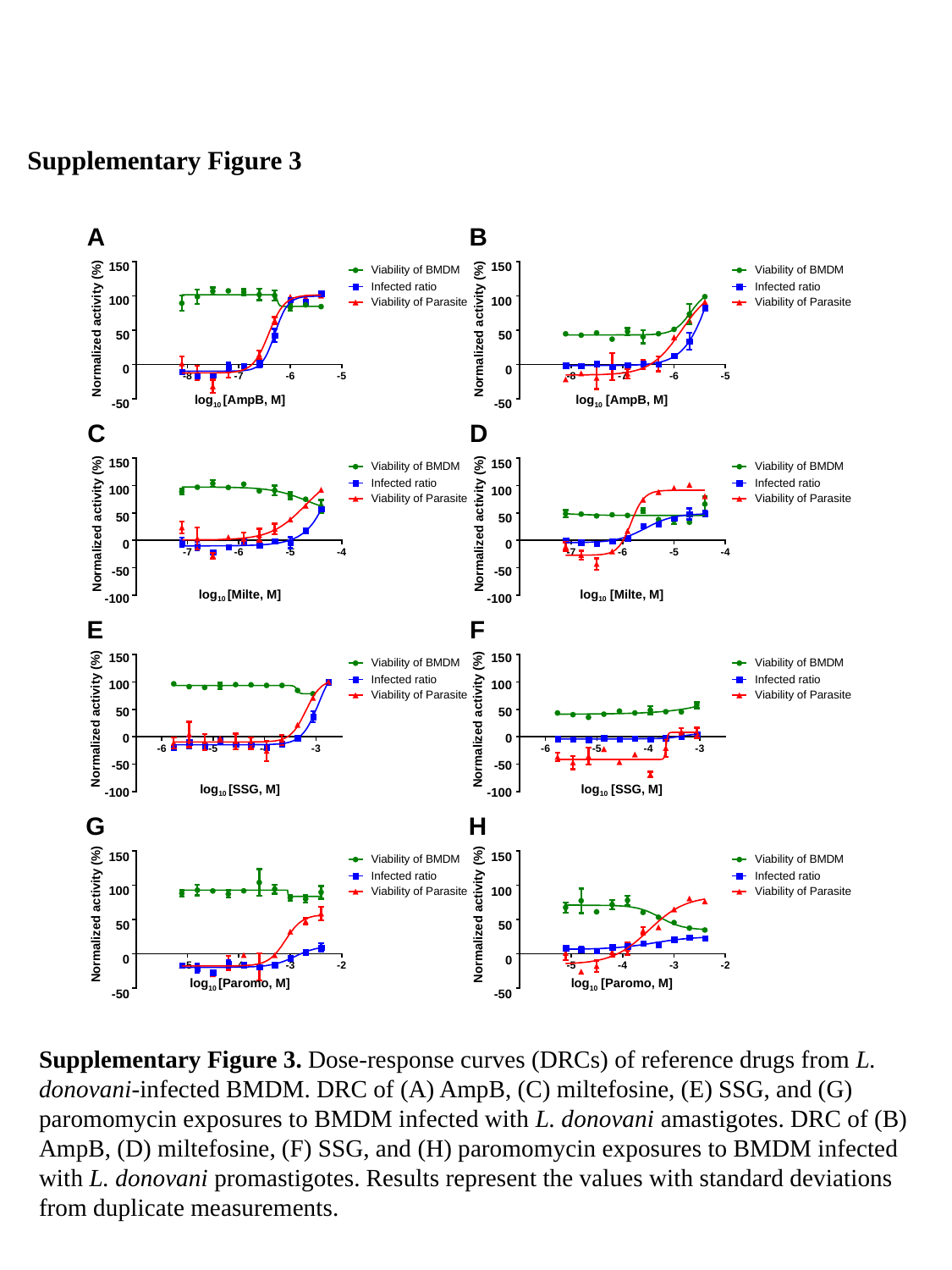

Supplementary Figure 3
A
B
150
100
50
0
-50
150
100
50
0
-50
Normalized activity (%)
Normalized activity (%)
log10 [AmpB, M]
log10 [AmpB, M]
C
D
150
100
50
0
-50
-100
150
100
50
0
-50
-100
Normalized activity (%)
Normalized activity (%)
log10 [Milte, M]
log10 [Milte, M]
E
F
150
100
50
0
-50
-100
150
100
50
0
-50
-100
Normalized activity (%)
Normalized activity (%)
log10 [SSG, M]
log10 [SSG, M]
G
H
150
100
50
0
-50
150
100
50
0
-50
Normalized activity (%)
Normalized activity (%)
log10 [Paromo, M]
log10 [Paromo, M]
Supplementary Figure 3. Dose-response curves (DRCs) of reference drugs from L. donovani-infected BMDM. DRC of (A) AmpB, (C) miltefosine, (E) SSG, and (G) paromomycin exposures to BMDM infected with L. donovani amastigotes. DRC of (B) AmpB, (D) miltefosine, (F) SSG, and (H) paromomycin exposures to BMDM infected with L. donovani promastigotes. Results represent the values with standard deviations from duplicate measurements.

## Slide 5
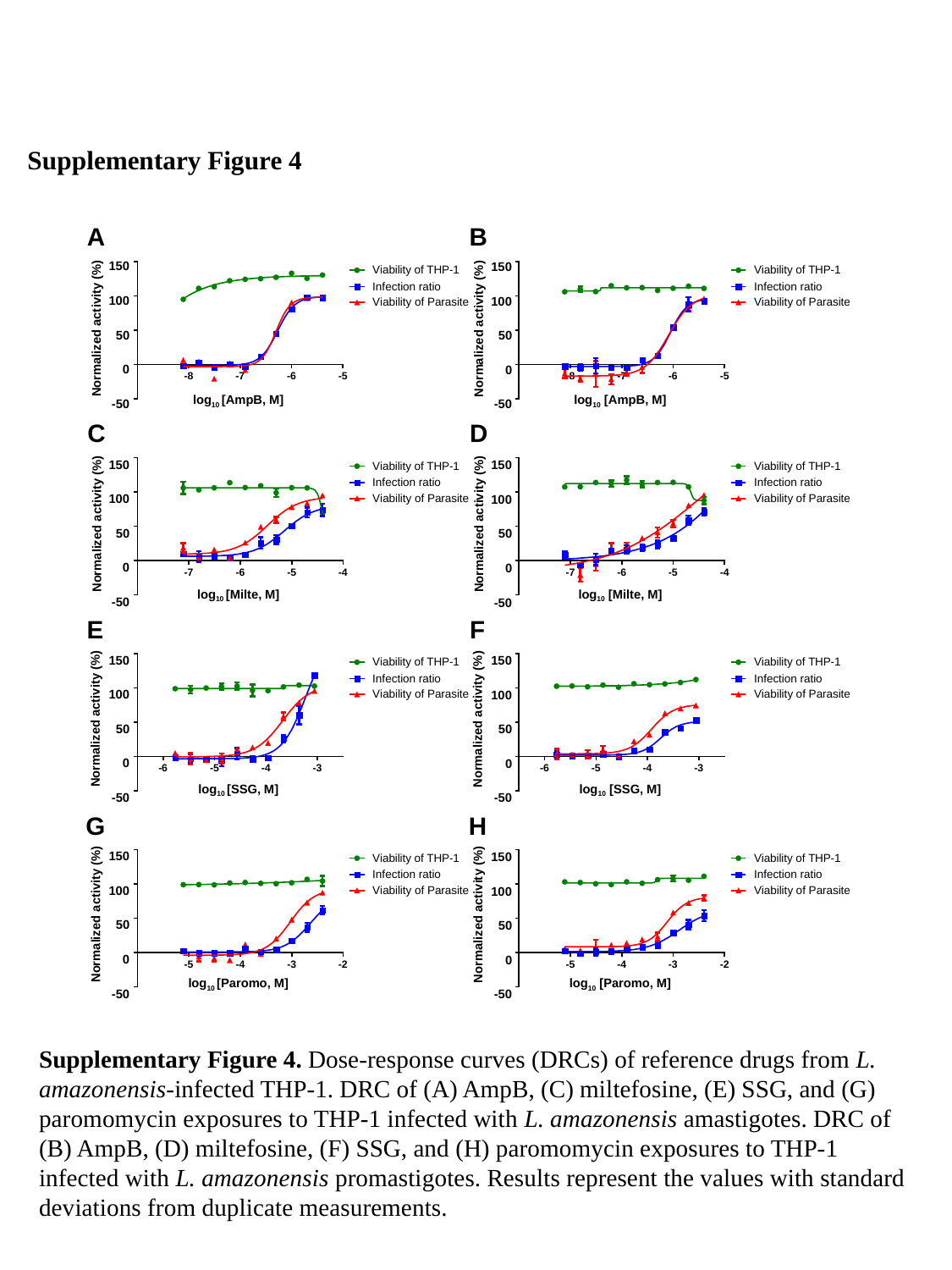

Supplementary Figure 4
A
B
150
100
50
0
-50
150
100
50
0
-50
Normalized activity (%)
Normalized activity (%)
log10 [AmpB, M]
log10 [AmpB, M]
C
D
150
100
50
0
-50
150
100
50
0
-50
Normalized activity (%)
Normalized activity (%)
log10 [Milte, M]
log10 [Milte, M]
E
F
150
100
50
0
-50
150
100
50
0
-50
Normalized activity (%)
Normalized activity (%)
log10 [SSG, M]
log10 [SSG, M]
G
H
150
100
50
0
-50
150
100
50
0
-50
Normalized activity (%)
Normalized activity (%)
log10 [Paromo, M]
log10 [Paromo, M]
Supplementary Figure 4. Dose-response curves (DRCs) of reference drugs from L. amazonensis-infected THP-1. DRC of (A) AmpB, (C) miltefosine, (E) SSG, and (G) paromomycin exposures to THP-1 infected with L. amazonensis amastigotes. DRC of (B) AmpB, (D) miltefosine, (F) SSG, and (H) paromomycin exposures to THP-1 infected with L. amazonensis promastigotes. Results represent the values with standard deviations from duplicate measurements.

## Slide 6
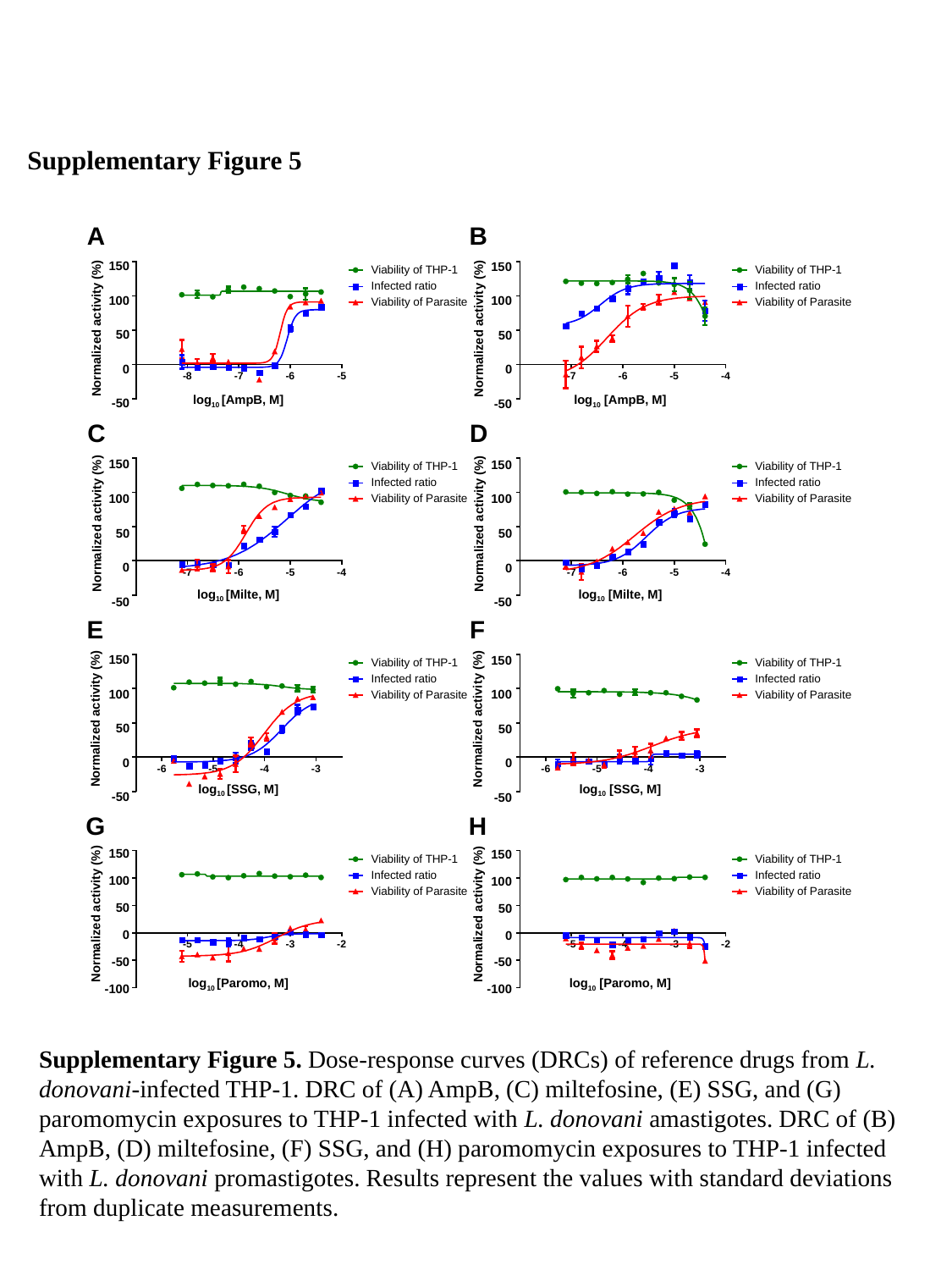

Supplementary Figure 5
A
B
150
100
50
0
-50
150
100
50
0
-50
Normalized activity (%)
Normalized activity (%)
log10 [AmpB, M]
log10 [AmpB, M]
C
D
150
100
50
0
-50
150
100
50
0
-50
Normalized activity (%)
Normalized activity (%)
log10 [Milte, M]
log10 [Milte, M]
E
F
150
100
50
0
-50
150
100
50
0
-50
Normalized activity (%)
Normalized activity (%)
log10 [SSG, M]
log10 [SSG, M]
G
H
150
100
50
0
-50
-100
150
100
50
0
-50
-100
Normalized activity (%)
Normalized activity (%)
log10 [Paromo, M]
log10 [Paromo, M]
Supplementary Figure 5. Dose-response curves (DRCs) of reference drugs from L. donovani-infected THP-1. DRC of (A) AmpB, (C) miltefosine, (E) SSG, and (G) paromomycin exposures to THP-1 infected with L. donovani amastigotes. DRC of (B) AmpB, (D) miltefosine, (F) SSG, and (H) paromomycin exposures to THP-1 infected with L. donovani promastigotes. Results represent the values with standard deviations from duplicate measurements.

## Slide 7
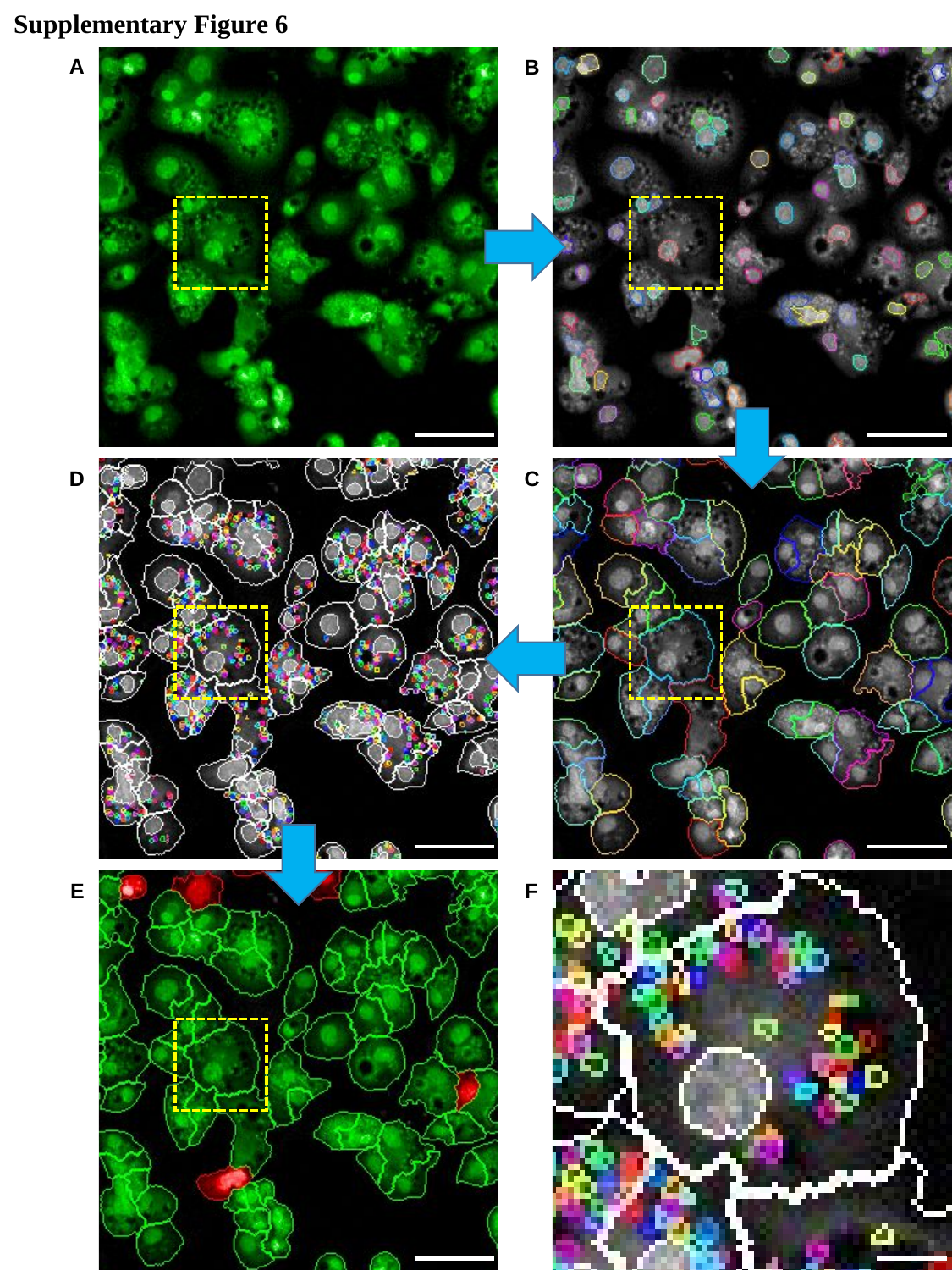

Supplementary Figure 6
A
B
D
C
E
F

## Slide 8
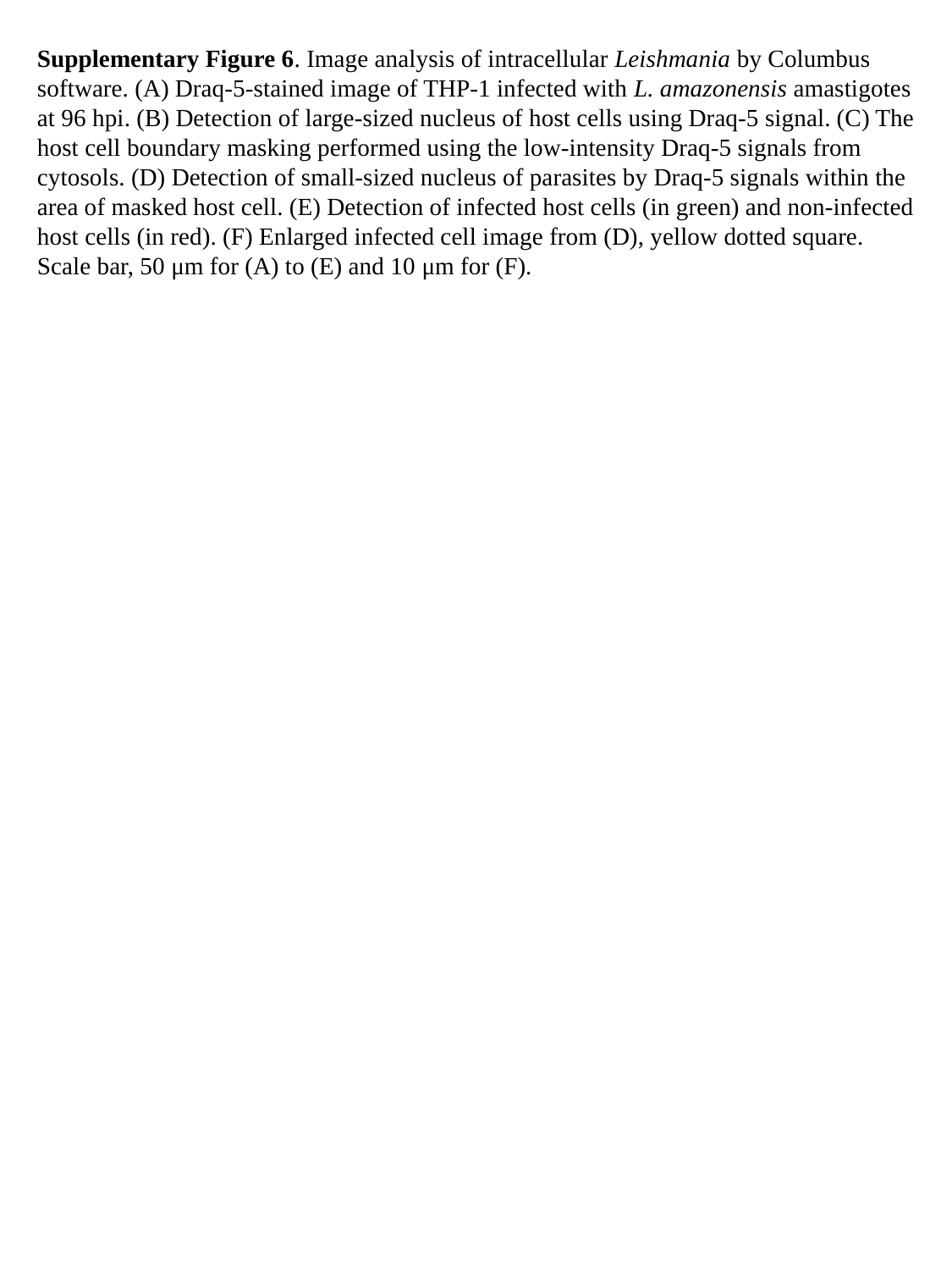

Supplementary Figure 6. Image analysis of intracellular Leishmania by Columbus software. (A) Draq-5-stained image of THP-1 infected with L. amazonensis amastigotes at 96 hpi. (B) Detection of large-sized nucleus of host cells using Draq-5 signal. (C) The host cell boundary masking performed using the low-intensity Draq-5 signals from cytosols. (D) Detection of small-sized nucleus of parasites by Draq-5 signals within the area of masked host cell. (E) Detection of infected host cells (in green) and non-infected host cells (in red). (F) Enlarged infected cell image from (D), yellow dotted square. Scale bar, 50 μm for (A) to (E) and 10 μm for (F).
