# Supplementary material for: Infectivity and Drug Susceptibility Profiling of Different Leishmania-Host Cell Combinations
Source: Pathogens. 2020 May 20;9(5):393. doi: 10.3390/pathogens9050393 (PMC7281264; doi:10.3390/pathogens9050393)
Supplement: Supplementary file 1 [file pathogens-09-00393-s001.zip › LeishHostDrug_Tables_Revision.DOCX]

| **Host cell** | ***Leishmania* for infection** | | **Infection ratio (IR)** | | | | | | | | | | | | | | |
| --- | --- | --- | --- | --- | --- | --- | --- | --- | --- | --- | --- | --- | --- | --- | --- | --- | --- |
|  |  |  | **3 hpi** | | | **24 hpi** | | | **48 hpi** | | | **72 hpi** | | | **96 hpi** | | |
|  | **Species** | **Infection form** | **1:5** | **1:10** | **1:20** | **1:5** | **1:10** | **1:20** | **1:5** | **1:10** | **1:20** | **1:5** | **1:10** | **1:20** | **1:5** | **1:10** | **1:20** |
| BMDM | *L. amazonensis* | ama | 0.857 | 0.934 | 0.951 | 0.936 | 0.969 | 0.974 | 0.966 | 0.972 | 0.977 | 0.959 | 0.971 | 0.979 | 0.973 | 0.985 | 0.986 |
|  |  | pro | 0.594 | 0.743 | 0.899 | 0.816 | 0.908 | 0.971 | 0.878 | 0.952 | 0.982 | 0.877 | 0.957 | 0.985 | 0.936 | 0.969 | 0.987 |
|  | *L. donovani* | ama | 0.758 | 0.813 | 0.798 | 0.615 | 0.750 | 0.709 | 0.644 | 0.721 | 0.740 | 0.701 | 0.790 | 0.809 | 0.662 | 0.734 | 0.793 |
|  |  | pro | 0.388 | 0.488 | 0.683 | 0.564 | 0.690 | 0.772 | 0.640 | 0.664 | 0.673 | 0.624 | 0.733 | 0.741 | 0.575 | 0.745 | 0.740 |
| THP-1 | *L. amazonensis* | ama | 0.522 | 0.704 | 0.840 | 0.747 | 0.811 | 0.898 | 0.767 | 0.848 | 0.913 | 0.798 | 0.874 | 0.909 | 0.805 | 0.893 | 0.915 |
|  |  | pro | 0.557 | 0.682 | 0.791 | 0.702 | 0.794 | 0.871 | 0.729 | 0.808 | 0.879 | 0.749 | 0.823 | 0.888 | 0.780 | 0.840 | 0.894 |
|  | *L. donovani* | ama | 0.825 | 0.904 | 0.949 | 0.872 | 0.910 | 0.949 | 0.880 | 0.944 | 0.965 | 0.905 | 0.948 | 0.970 | 0.880 | 0.924 | 0.933 |
|  |  | pro | 0.559 | 0.638 | 0.736 | 0.678 | 0.780 | 0.869 | 0.765 | 0.779 | 0.899 | 0.735 | 0.742 | 0.918 | 0.677 | 0.786 | 0.943 |

**Supplementary Table 1**. Infection ratio (IR) of host cells infected with *L.* *amazonensis* or *L. donovani* by multiplicity of infection (MOI). Results represent the values with standard deviations from quadruplicate measurements (ama = amastigote, pro = promastigote).

| **Host cell** | ***Leishmania* for infection** | | **The number of parasites per cell (P/ϕ)** | | | | | | | | | | | | | | |
| --- | --- | --- | --- | --- | --- | --- | --- | --- | --- | --- | --- | --- | --- | --- | --- | --- | --- |
|  |  |  | **3 hpi** | | | **24 hpi** | | | **48 hpi** | | | **72 hpi** | | | **96 hpi** | | |
|  | **Species** | **Infection form** | **1:5** | **1:10** | **1:20** | **1:5** | **1:10** | **1:20** | **1:5** | **1:10** | **1:20** | **1:5** | **1:10** | **1:20** | **1:5** | **1:10** | **1:20** |
| BMDM | *L. amazonensis* | ama | 5.37 | 9.56 | 15.9 | 10.5 | 14.9 | 20.3 | 15.3 | 18.4 | 23.1 | 17.2 | 19.6 | 23.6 | 19.2 | 22.9 | 23.9 |
|  |  | pro | 2.81 | 3.72 | 7.59 | 5.25 | 7.69 | 14.0 | 7.78 | 11.3 | 17.7 | 9.50 | 13.3 | 18.4 | 12.1 | 15.7 | 19.5 |
|  | *L. donovani* | ama | 3.36 | 4.47 | 5.11 | 2.98 | 4.20 | 5.03 | 3.69 | 4.82 | 5.90 | 4.94 | 6.60 | 7.97 | 4.49 | 5.80 | 6.60 |
|  |  | pro | 1.68 | 1.96 | 2.83 | 2.49 | 3.42 | 5.14 | 3.00 | 4.12 | 5.34 | 3.20 | 4.64 | 5.65 | 3.50 | 5.06 | 6.13 |
| THP-1 | *L. amazonensis* | ama | 3.34 | 5.25 | 8.41 | 6.19 | 8.36 | 13.0 | 7.55 | 10.4 | 15.7 | 9.07 | 12.6 | 16.3 | 9.24 | 13.2 | 17.0 |
|  |  | pro | 2.56 | 3.21 | 4.42 | 3.96 | 5.51 | 8.19 | 4.43 | 6.00 | 8.32 | 4.89 | 6.38 | 8.87 | 5.15 | 6.62 | 9.15 |
|  | *L. donovani* | ama | 4.23 | 6.87 | 10.1 | 5.83 | 8.29 | 13.5 | 6.50 | 10.3 | 16.0 | 7.79 | 12.6 | 19.4 | 7.91 | 12.6 | 17.1 |
|  |  | pro | 2.40 | 2.62 | 3.23 | 3.31 | 4.20 | 5.60 | 4.12 | 4.28 | 6.53 | 3.83 | 3.89 | 7.10 | 3.38 | 4.53 | 8.72 |

**Supplementary Table 2**. The number of parasites per cell (P/φ) of host cells infected with *L.* *amazonensis* or *L. donovani* by multiplicity of infection (MOI). Results represent the values with standard deviations from quadruplicate measurements (ama = amastigote, pro = promastigote).

| **Host cell** | ***Leishmania* for infection** | | **EC_50_ value of drugs in μM**  **(95% confidence intervals)** | | | |
| --- | --- | --- | --- | --- | --- | --- |
|  | **Species** | **Infection form** | **Amp B** | **Milte** | **SSG** | **Paromo** |
| BMDM | *L. amazonensis* | ama | 0.524  (0.489-0.561) | 16.4  (15.7-17.1) | 465  (426-507) | >4000 |
|  |  | pro | 0.832  (0.746-0.927) | 60.1  (52.1-69.4) | 745  (671-826) | 1371  (961-1955) |
|  | *L. donovani* | ama | 0.505  (0.475-0.536) | 44.3  (32.1-61.0) | >1000 | >4000 |
|  |  | pro | 2.56  (2.36-2.78) | 2.81  (2.33-3.38) | >1000 | >4000 |
| THP-1 | *L. amazonensis* | ama | 0.536  (0.504-0.569) | 12.4  (9.62-16.0) | 596  (391-907) | 3289  (2944-3673) |
|  |  | pro | 0.887  (0.817-0.964) | 32.0  (16.9-60.5) | 940  (671-1315) | >4000 |
|  | *L. donovani* | ama | 0.877  (0.826-0.932) | 8.53  (5.12-14.2) | 218  (158-301) | >4000 |
|  |  | pro | 0.366  (0.178-0.749) | 2.89  (2.46-3.40) | >1000 | >4000 |

**Supplementary Table 3**. Drug susceptibility (EC_50_) against anti-leishmanial reference drugs in each host cell-parasite pair; EC_50_ values calculated based on infection ratios (IR). Results represent the values with standard deviations from duplicate measurements (ama = amastigote, pro = promastigote).

| **Host cell** | ***Leishmania* for infection** | | **EC_50_ value of drugs in μM**  **(95% confidence intervals)** | | | |
| --- | --- | --- | --- | --- | --- | --- |
|  | **Species** | **Infection form** | **Amp B** | **Milte** | **SSG** | **Paromo** |
| BMDM | *L. amazonensis* | ama | 0.420  (0.389-0.453) | 8.49  (6.94-10.4) | 292  (242-353) | >4000 |
|  |  | pro | 0.818  (0.746-0.898) | 18.9  (16.1-22.3) | 270  (240-305) | 1346  (954-1899) |
|  | *L. donovani* | ama | 0.381  (0.341-0.425) | 15.7  (10.3-24.0) | 638  (525-776) | 1854  (1385-2481) |
|  |  | pro | 1.40  (0.873-2.25) | 1.47  (1.34-1.60) | >1000 | 325  (244-434) |
| THP-1 | *L. amazonensis* | ama | 0.495  (0.450-0.545) | 3.82  (3.23-4.51) | 211  (176-253) | 1069  (931-1228) |
|  |  | pro | 0.815  (0.703-0.944) | 9.57  (4.20-21.8) | 191  (157-231) | 1156  (924-1446) |
|  | *L. donovani* | ama | 0.640  (0.547-0.748) | 1.37  (1.22-1.53) | 98.9  (78.6-124) | >4000 |
|  |  | pro | 0.493  (0.325-0.747) | 1.71  (1.33-2.21) | >1000 | >4000 |

**Table 1**. Drug susceptibility (EC_50_) against anti-leishmanial reference drugs in each host cell-parasite pair; EC_50_ values calculated based on the number of parasites per cell (P/φ). Results represent the values with standard deviations from duplicate measurements (ama = amastigote, pro = promastigote).
